# Paneth cell development in the neonatal gut: pathway regulation, development, and relevance to necrotizing enterocolitis

**DOI:** 10.3389/fcell.2023.1184159

**Published:** 2023-05-17

**Authors:** Jiahui Yang, Yongyan Shi

**Affiliations:** Department of Pediatrics, Shengjing Hospital of China Medical University, Shenyang, China

**Keywords:** neonatal necrotizing enterocolitis, Paneth cells, gut microbiota, intestinal stem cells, necroptosis, apoptosis, pyroptosis

## Abstract

Paneth cells (PCs) are intestinal epithelial cells (IECs) that contain eosinophilic granules, which are located in Lieberkühn crypts. An increasing number of animal and human experiments have indicated that PCs are involved in the progression of a variety of intestinal as well as systemic inflammatory responses including necrotizing enterocolitis (NEC). NEC is an enteric acquired disease with high mortality that usually occurs in premature infants and neonates, however the underlying mechanisms remain unclear. In this review, we summarize the features of PCs, including their immune function, association with gut microbiota and intestinal stem cells, and their mechanism of regulating IEC death to explore the possible mechanisms by which PCs affect NEC.

## 1 Introduction

Necrotizing enterocolitis (NEC) is one of the most common acute intestinal necrotizing diseases (Blakely et al., 2021). In neonatal intensive care units, NEC has high morbidity and mortality rates, with 90%–95% of NEC cases occurring in premature infants and few in full-term infants (Horbar et al., 2012; Patel et al., 2015; Battersbyet al. 2018). After 30 years of intensive research, the exact cause of NEC is still unknown, but premature infants with immature intestinal development, imbalanced intestinal microbiota, and imbalanced inflammatory responses, as well as hypoxia, artificial feeding, and other factors, are associated with a high risk of developing NEC (Niño et al., 2016; Caplan et al., 2019; Masi et al., 2021). It is necessary to explore the pathogenesis and therapeutic targets of NEC to reduce neonatal mortality rate and improve the quality of life of premature infants.

**FIGURE 1:**
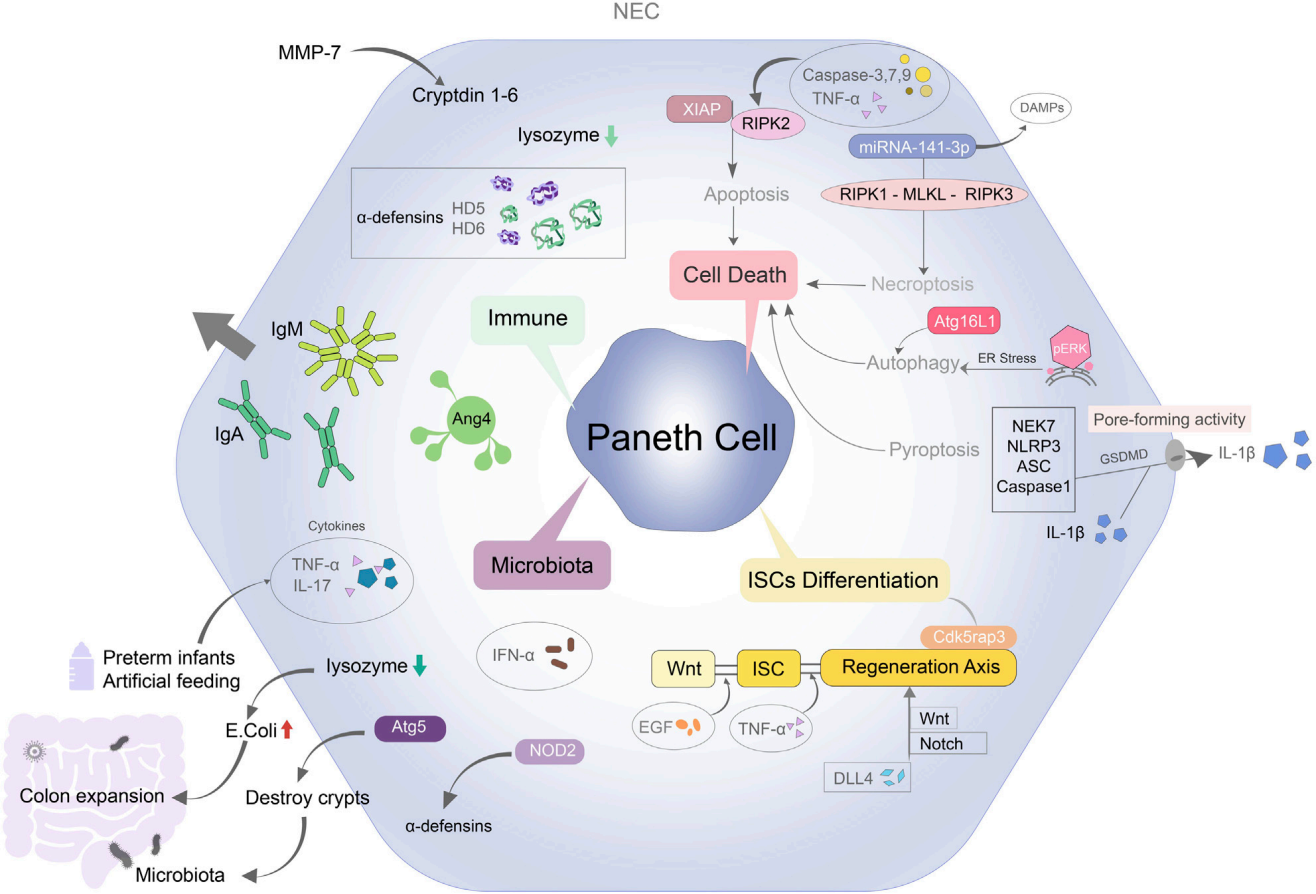
Overview of mechanisms regulating Paneth cell leads to NEC. PC can contribute to NEC through immune system, microbiota, cell deaths and intestinal stem cells differentiation. PC protects immune barrier function by secreting specific antimicrobial peptides such as lysozyme and defensins. The inflammatory factors in the preterm infants and artificial feeding infants with NEC are increased. Lysozyme secreted by PCs is decreased, the structure of intestinal crypts is destroyed, and a large number of *Escherichia coli* colonize in the intestinal tract, which aggravates the degree of colon expansion. Disruption of the functional structure of PCs also affects Wnt and Notch signaling mediated intestinal stem cell differentiation. PC-mediated apoptosis, autophagy, necroptosis and pyroptosis are also closely related to NEC.

One possible explanation may be suggested by Paneth cell (PC), a highly specific cell of the small intestinal, which have the function of coordinating the physiological environment of the small intestine. The history of this special type of intestinal columnar epithelial cell can be traced back to a century ago when it was first documented by Gustav Schwalbe and detailed in 1888 by Josef Paneth in Vienna (Paneth, 1887). The identity of the content of PC was still largely unknown. Not until 1967, lysozyme, as a constituent of the PC granules, was found in mouse intestinal mucosa (Deckx et al., 1967). A large number of antibacterial substances and proteins contained in PC play an important role in maintaining the host-microbial relationship, maintaining intestinal microbial balance, regulating intestinal stem cell development and immune protection (Clevers and Bevins, 2013) (Figure 1).

In this review, we explore the relationship between PCs and intestinal epithelial barrier, how PCs affect intestinal stem cell differentiation and gut microbiota ecological balance, and whether several modes of intestinal epithelial death contribute to NEC by affecting PCs function.

## 2 The characteristics and biological role of PC

PCs are highly specific cells of the small intestine, located within Lieberkühn crypts in a pyramidal shape (Paneth, 1887). Under normal conditions, PCs are located all along the entire length of the small intestine, but under disease conditions, they may also be found in the esophagus and colon (Chen et al., 2015; Singh et al., 2020; Gleizes et al., 2021). PCs first appear at 13.5 weeks of gestation, and at this point, their density is very low (Stanford et al., 2020). As gestational age increases, the number of PCs gradually increase until they reach maturity at term (Kandasamy et al., 2014). Preterm infants are prone to abnormal colonization of intestinal microorganisms and enrichment of special metabolites within a few weeks after birth (Korpela et al., 2018; Samara et al., 2022). During this period, PCs are not mature and anti-inflammatory factor levels are insufficient; hence, they cannot play a role in defending against pathogen invasion and protecting the intestinal barrier, increasing the susceptibility to NEC (Autran et al., 2018; Lueschow et al., 2018; Li et al., 2020).

**FIGURE 2:**
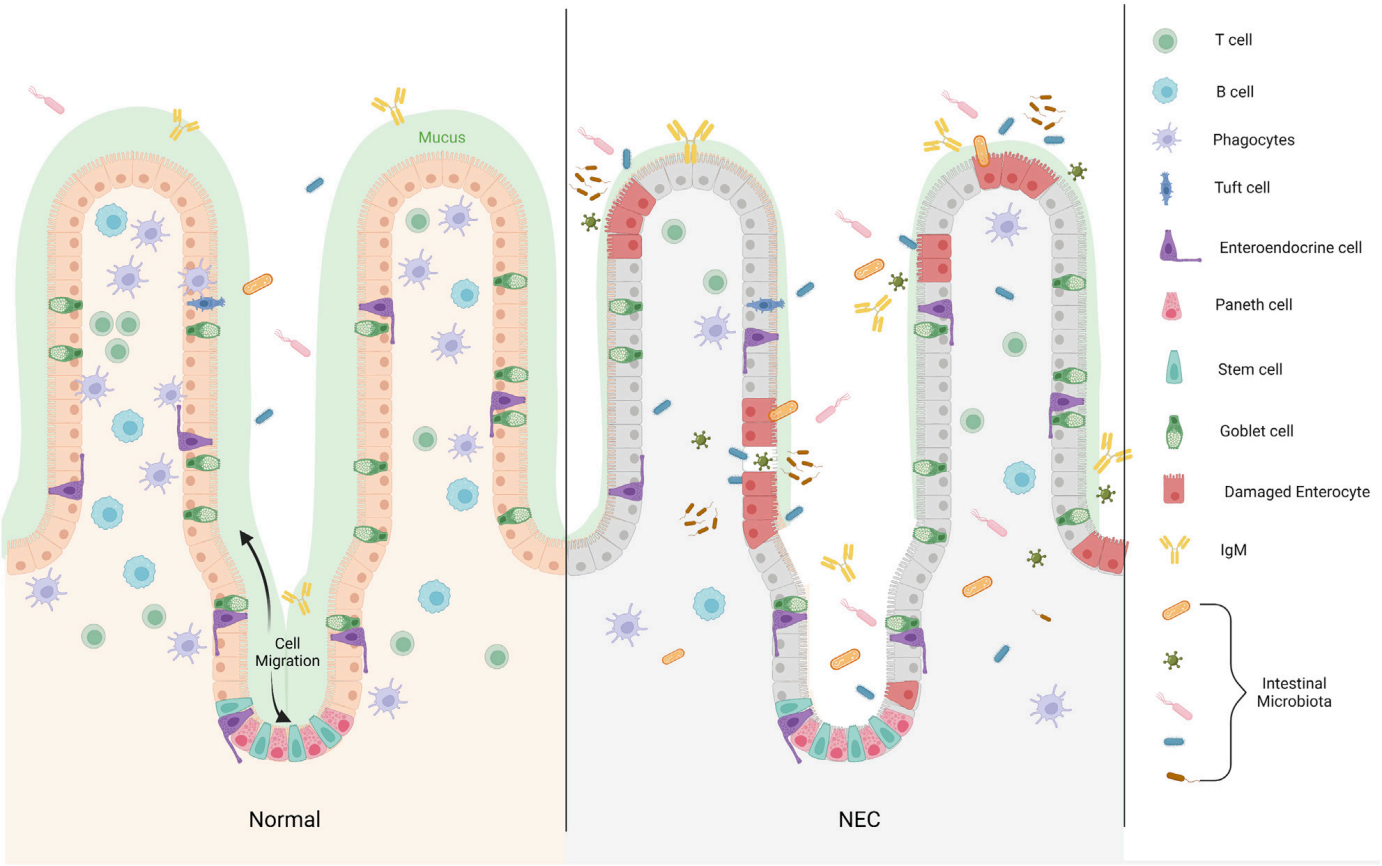
The distribution of Paneth cell in epithelium of the small intestine. Differentiated cells migrate from the base of the crypt to the villus, whereas PCs remain at the base of the crypt and are distributed between Lgr5+ stem cells. In the development of NEC, the microbial dysbiosis decrease binding of bacteria by IgA, and the mucosal layer becomes thinner. Increased inflammatory infiltration reduce the secretion of immune cells in the intestinal lumen, which is not enough to resist the invasion of pathogenic microorganisms. Large numbers of microbiota pass through the disrupted intestinal barrier (Created with BioRender.com).

Most of the functions of PCs are related to their secreted products. Specific antibacterial products, lysozyme, and a variety of antimicrobial peptides in PCs can form an immune barrier near the crypt epithelium to resist the invasion of pathogenic microorganisms (Yu S. et al., 2020; Azabdaftari and Uhlig, 2021). By secreting Wnt, Notch, EGF, and other signals, PCs form a “stem cell niche” with intestinal stem cells (ISCs) in the intestinal crypts, regulate the proliferation and differentiation of ISCs and self-renewal of intestinal epithelial cells (IECs), and maintain homeostasis of the mechanical barrier of the intestinal mucosa (Yu et al., 2018; van Es et al., 2019; Böttcher et al., 2021; Weis et al., 2021) (Figure 2).

## 3 PCs and immune regulation

The intestinal barrier is an important defense against harmful substances and pathogenic microorganisms found in the external environment. It is composed of four parts: the biological barrier composed of intestinal flora, the chemical barrier composed of the mucus layer, the mechanical barrier composed of epithelial cells, and the immune barrier composed of immune cells and immune factors (Atarashi et al., 2015; Moore et al., 2016; Sun et al., 2018; Doron et al., 2021). Intestinal homeostasis depends on the combined regulation of immune, mechanical, and biological barriers, which PCs play a major role in the maintenance of these three barriers.

PCs secrete antimicrobial peptides and proteins (AMPs), enzymes and growth factors, which form a biochemical barrier near the crypts, prevent bacterial invasion, and maintain stability of the intestinal environment (Brodrick et al., 2011; Gubatan et al., 2021; He et al., 2022). These defense proteins are released into the intestinal lumen as “degranulation” by cytoplasmic granules at the top of PC(Ayabe et al., 2000).

AMPs fall into two major classes in the human body: cathelicidins and defensins (Liang et al., 2022). During the neonatal period, cathelicidins play an important role in the maintenance of environmental homeostasis in epithelial cells (Chen et al., 2021; Gubatan et al., 2021). As time goes by, immature PCs are gradually replaced by mature PCs, while cathelicidins also gradually decrease and eventually disappear (Ménard et al., 2008). The protective effect of defensin in the intestinal barrier is dominant (Fusco et al., 2021). The transition of AMPs from one to the other is probably around the second trimester of human pregnancy (between 20 and 28 weeks) and 10–21 days after birth in mice, which highly coincides with the onset of NEC (Mallow et al., 1996; Hu et al., 2019).

The main defensin secreted by PCs is α-defensin (also known as cryptdins in mice) (Ayabe et al., 2002). The human defensin (HD)5 and HD6 have indirect antiviral effects owing to their similarity to viral coats and their ability to affect DNA and RNA replication (Bevins, 2013; Ehmann et al., 2019). HD5 can also be used as an effective marker to identify PCs (Heida et al., 2016). Before 28 weeks of gestation, HD5 expression is correlated with the number of PCs and gradually increases. Between weeks 29 and 37, the number of PCs and the level of HD5 rapidly increase until the expression of HD5 is no longer correlated with the number of PCs (McElroy et al., 2013). The clinical peak of NEC incidence occurs at 29–33 weeks of corrected gestational age, which coincides with the rapid growth of PCs and expression of HD5 (Ehmann et al., 2019). The defense proteins secreted by PCs are overactivated in the immature immune system, resulting in the occurrence of inflammation and colonization by pathogenic bacteria in the immature intestinal tract, ultimately leading to the occurrence of NEC (González-Rivera et al., 2011; McElroy et al., 2013).

Lysozyme is considered to be an indicator of PC localization (Peeters and Vantrappen, 1975). Lysozyme can destroy microbial cell membranes and regulate immune function (Bel and Hooper, 2018). Lysozyme achieves bactericidal action by processing the bacterial cell wall and destroying the permeability of the bacterial cell membrane (Bel et al., 2017). In the absence of PCs, the secretion of specific antibacterial substances, such as defensins and lysozyme, is reduced, leading to the inability to resist the invasion by foreign pathogens and microorganisms (Schaart et al., 2009). When the intestinal barrier is destroyed, more pathogens enter the intestine, and break the serious imbalance of the intestinal environment (Bergmann et al., 2013). This suggests that the relative PC deficiency was the key to the intestinal inflammation.

The immunological changes of NEC include the deficiency of anti-inflammatory mediators and the increase of pro-inflammatory mediators, most of which are produced by PCs. Secretory immunoglobulin (Ig)A and M are produced at the apex of PCs. Secretory IgA is released through hydrolysis of the apical part of the PC membrane, which inhibits the adhesion and adsorption of pathogens (viruses and bacteria) in the intestinal mucosa that form the biofilm, thereby protecting the mucosal barrier (Wang et al., 2017). PCs are also an important source of cathepsin G, which sterilizes the intestinal epithelium, regulates the immune response, and destroys pathogens (Zamolodchikova et al., 2020). When intestinal mucosal inflammation occurs, PCs are stimulated by microorganisms to release a large amount of lysozyme and cathepsin G, which kill harmful pathogens and mediate homeostasis of the intestinal flora and intestinal biological barrier (Burclaff et al., 2022).

PCs also produce the pro-inflammatory tumor necrosis factor-α (TNF-α), which accumulates in secretory granules and enhances systemic inflammation (Tan et al., 1993). When PCs are destroyed, TNF-α is released, resulting in increased diffusion of IECs (Bykov, 2014). Interleukin-17 (IL-17) is another anti-inflammatory factor secreted by PCs, which co-reacts with TNF-α in acute inflammation to participate in the rapid response to systemic inflammatory factors (Gyongyosi et al., 2019). IL-17A is normally produced by activated T lymphocytes (Park et al., 2005). However, production of IL-17A in PCs is induced in contrast to TNF-α secretion, and increases when the intestinal is infected (Bykov, 2014). Therefore, the content of anti-inflammatory mediators in PCs decreases, and the increase in the content of pro-inflammatory mediators can be used as one of the evaluation indicators of NEC.

## 4 PCs and impaired differentiation of ISCs

ISCs, as an important component of environmental homeostasis in the gut, are characterized by the expression of the stem cell marker Lgr5^+^(Tian et al., 2011). Differentiated cells migrate from the base of the crypt to the villus, whereas PCs remain at the base of the crypt and are distributed between Lgr5+ stem cells, providing ligands such as Wnt, Notch, epidermal growth factor (EGF), TGF-α to ISCs(Sato et al., 2011).

As an important factor in ISC differentiation, Wnt signaling regulates ISC lineage differentiation by activating the β-catenin/Tcf transcription program and regulating the activity of mitogen-activated protein kinase (Zha et al., 2019; Wang et al., 2020; Wang et al., 2021; Li et al., 2023). Cell-specific knockout of PC *in vivo* resulted in the loss of Lgr5+ stem cells (Pentinmikko et al., 2019). In both mouse NEC models and clinical NEC children, ISC expression, Wnt activity and intestinal cell differentiation were downregulated (Li et al., 2019). Notch is an inhibitor of Wnt: when the Wnt level increases or the Notch level decreases, the signal is transmitted to ISCs and stimulates the continuous renewal of PCs(Pentinmikko et al., 2019). The expression of Notch signaling is very low under normal conditions (Sancho et al., 2015). When the intestine is injured, Notch signaling is activated to stimulate the release of ISCs ligands from PCs which plays a role in maintaining the intestinal homeostasis (Yu et al., 2018; Jones et al., 2019). Notch ligand DLL4 binds to Notch1 and Notch2 to promote the homeostasis of the ISC microenvironment as well as the growth and development of ISCs (Dominguez-Brauer et al., 2016).

ISCs are essential for maintaining intestinal homeostasis and protecting the neonatal intestine (Hua et al., 2012; Zou et al., 2018). Intestinal epithelial cell lineages are damaged by stressors such as ischemia, hypoxia, and low temperature, and ISCs are responsible for promoting the repair and regeneration of IEC lineages damaged by these stressors (Lee C. et al., 2018; Holmberg et al., 2018; Sittipo et al., 2021). The loss of Cdk5 regulatory subunit-related protein 3, which is secreted by PCs, can affect the cell cycle progression of ISCs, cause differentiation of ISCs into PCs and the proliferation of IECs (Quintero et al., 2021). Many studies have found that the number of PCs and ISCs is significantly reduced in NEC mice. When NEC and other inflammatory reactions occur, the amount of IL-22 (secreted by CD4+T cells) decreases, which can affect the differentiation and maturation of PCs (Lindemans et al., 2015). The secretion of Wnt and Notch signals in PCs is inhibited, which prevent the differentiation and maturation of ISC (Zha et al., 2019; Gaudino et al., 2021). These studies highlight the critical link between ISC function, PC-secreted ISC ligands, and intestinal barrier disruption in NEC.

## 5 PCs and imbalance of intestinal flora

The intestinal microbiota is important for regulating physiology, metabolism, and the host immune response. Intestinal homeostasis depends on the co-regulation of symbiotic bacteria, IECs, and mucosal immune cells. Maintaining the balance of intestinal microbiota is crucial for sustaining the host nutritional metabolism and immune system (Lee Y.-S. et al., 2018). Intestinal dysbiosis have been associated with a variety of diseases, including inflammatory bowel disease, NEC, sepsis, diabetes, and obesity (Wellman et al., 2017).

PC-derived α-defensin is the main bactericidal protein in the intestinal epithelium. α-defensin promotes homeostasis by establishing and maintaining the intestinal microbiota (Kamioka et al., 2022). A reduction in the levels of α-defensin leads to alterations in the composition of the intestinal microbiota, which leads to the occurrence of various diseases (Hayase et al., 2017; Takakuwa et al., 2019). α-defensin level is significantly reduced in patients with coding mutations in the nucleotide-binding oligomerization domain protein 2 (NOD2). These mutations are the basis of genetic susceptibility to a variety of intestinal diseases; therefore, the reduced expression of α-defensin in PCs may change the composition of the intestinal microbiota and lead to a series of related intestinal inflammatory reactions (Fakhoury et al., 2020).

The loss of PCs results in decreased levels of lysozymes, altered intestinal bacterial composition, increased abundance of *E. coli*, hypersensitivity of the viscera, the epithelial proliferation and anti-inflammatory macrophages in the intestinal (Yu T.-X. et al., 2020; Nakamura et al., 2021; Chiang et al., 2022). Oral lysozyme can prevent the excessive growth of *E. coli* and visceral hypersensitivity induced by maternal isolation, thus achieving the prevention and treatment of NEC (Riba et al., 2017). There are a variety of bacteria in the gut that interact during the development and progression of NEC. Intestinal bacterial infection causes massive bacterial replication and increased activity of specific organisms, leading to an imbalance in the metabolites of the intestinal environment and ultimately causing NEC (Morowitz et al., 2010). However, increased levels of NEC-related metabolites, such as some small bactericidal peptides, bacteriocin, firmicide (produced by *Clostridium perfringens* and *C. difficile*), encoded polypeptides, and butylactones, result in cell lysis and the release of immune-stimulating compounds, increasing the severity of NEC (Olm et al., 2019).

The developmental pattern of PCs causes exposure of premature infants to various external pathogenic factors before the developing PCs can reach their full potential (Kandasamy et al., 2014). Additionally, the disruption of the normal intestinal tract can induce NEC (White et al., 2017; Lueschow et al., 2018; Lueschow et al., 2020). Exogenous factors also affect the gut microbiota. Murine norovirus causes increased interferon (IFN)-γ expression, intestinal microbiome-mediated inflammation, the loss of PCs, and bacterial dysregulation in mouse intestinal immunopathology (Farin et al., 2014; Rocha-Pereira et al., 2018). Loss of the specific autophagy protein ATG5 in PCs destroys intestinal crypts and causes excessive intestinal inflammation (Burger et al., 2018). When premature exposure to exogenous antigens occurs (for example, during formula feeding), the levels of inflammatory cytokines increase, and the resulting anti-inflammatory environment results in the formation of fewer PCs with compromised functional capacity (Qi-Xiang et al., 2022). This environment leads to reduced levels of adenosine phosphate, affecting the differentiation process of stem cells. The loss of intestinal epithelial cells makes the intestinal barrier unable to resist the invasion of inflammatory factors and intestinal microorganisms, which ultimately contributes to NEC (Autran et al., 2018; Battistini et al., 2020; Lueschow et al., 2020; Nolan et al., 2020).

## 6 PCs and IEC death

The death modes of intestinal cells include apoptosis, autophagy, necrosis, necroptosis, and pyroptosis. The main pathological change that occurs during NEC is intestinal tissue necrosis, and many different cell death pathways are related to the pathogenesis of NEC. PCs may participate in the pathogenesis of NEC by regulating multiple types of intestinal cell death.

### 6.1 Apoptosis

Apoptosis is a common mode of death in normal cells that manifests as cell pseudopodia contraction, cell pyknosis, fragmentation, dissolution, and plasma membrane blistering (Günther et al., 2013). Some genes and protein products related to cell proliferation and apoptosis are related to the development of PCs and NEC.

Although apoptosis is not an extremely important mechanism for maintaining intestinal homeostasis, maladjusted or excessive apoptosis can seriously impair intestinal physiology. In mice with an elevated apoptosis phenotype of the IECs, spontaneous intestinal inflammation or increased sensitivity to intestinal inflammation occurs (Li et al., 2018). Specific deletion of the gene *NEMO* (nuclear factor-κB (NF-κB) basic regulator) in IECs leads to intraepithelial TNF-dependent apoptosis, followed by intestinal barrier rupture and bacterial translocation into the intestinal wall, triggering a cellular inflammatory response (Nenci et al., 2007).

The occurrence of intestinal inflammation is related to the disappearance of antibiotics and the destruction of intestinal barrier. X-linked inhibitor of apoptosis protein (XIAP), which is a key component of the NOD1 and NOD2 complex, is a necessary ubiquitin ligase for NOD2 signaling (Wang et al., 2017). It is recruited into the NOD complex via receptor kinase 2 (RIPK2), ubiquitinated RIPK2, and the recruitment of linear ubiquitin chain assembly complexes linking NOD-mediated bacterial recognition with NF-κB activation and IL secretion (Larabi et al., 2020). Additionally, XIAP is an inhibitor of non-immunogenic apoptotic cell death pathways; it directly binds and inhibits caspase-3, -7, and -9, thus limiting apoptotic cell death (Damgaard et al., 2012; Damgaard et al., 2013). XIAP does not influence the intestinal microbiota but is essential for maintaining homeostasis. The levels of bactericidal substances, such as defensin and lysozyme, were significantly reduced in the ileum of XIAP-knockout mice, which suggests a deficiency of PCs(Wahida et al., 2021). XIAP reduces levels of inflammatory cytokines such as IL-1 and TNF-α; thus, the loss of XIAP may contribute to the loss of TNF receptor-1-dependent PCs (Azabdaftari and Uhlig, 2021; Strigli et al., 2021). Furthermore, loss of XIAP reduces toll-like receptor 5 and TNF signaling in myeloid cells, resulting in defects or disorders in the PCs and myeloid cells, contributing to IEC apoptosis (Wahida et al., 2021). The significantly increased inflammatory IL, TNF-α and caspase-3 in intestinal epithelial cells during autophagy can cause mucosal injury by increasing intestinal mucosal permeability, which is also important in NEC (Zhou et al., 2017).

### 6.2 Necroptosis

Necroptosis is a special mode of death that exhibits similarities with both apoptosis and necrosis. The cytological morphology of necroptosis is similar to that of traditional necrosis, which is a highly inflammatory cell death. During necroptosis, RIPK1 recruits and activates RIPK3, which phosphorylates mixed-lineage kinase domine-like protein (MLKL), leading to MLKL oligomerization and transfer to the cell membrane; MLKL then depolarizes the cell membrane, leading to cell rupture (Liu et al., 2021). RIPK1 is a major regulator of IEC survival, homeostasis, and inflammation. Specific knockdown of the RIPK1 in IECs can contribute to apoptosis, villus atrophy, and loss of PCs (Dannappel et al., 2014).

Necroptosis may be an important form of NEC-related IEC death. The expression of RIPK1, RIPK3, and MLKL in the intestinal mucosa of children with NEC was significantly upregulated, and this upregulation correlated with the severity of NEC (Werts et al., 2020). Intestinal epithelial cell-specific caspase-8-deficient mice have significantly increased necroptosis levels, resulting in a decrease in the number of PCs, showing progressive ileocecal inflammatory lesions and a high susceptibility to colitis, indicating that PC necroptosis is involved in the occurrence of intestinal inflammation (Sanders, 2005; Nenci et al., 2007; Schwarzer et al., 2020). When programmed necroptosis occurs, injury-related molecular patterns in cells are released, resulting in persistent inflammation and secondary tissue damage, which reduces the secretion of antimicrobial peptides and lysozyme and weakens the defense function against pathogens (Bertheloot et al., 2021). The high expression level of RIPK1/RIPK3 in human and mouse PCs suggests that PCs have a unique sensitivity to necroptosis (Duan et al., 2022). PC necroptosis-induced intestinal inflammation is consistent with the clinical characteristics of NEC in preterm infants, severe intestinal mucosal necrosis, and severe systemic inflammatory response, suggesting that PC necroptosis may be an important event in the pathogenesis of NEC.

Previous studies have reported necroptosis of IECs in NEC mice and that inhibiting necroptosis of IECs could reduce inflammation in these mice. We further found necroptosis of PCs in the intestinal crypts in NEC mice (Shi et al., 2018; Liu et al., 2022). When PCs experience programmed necrosis, resistance to pathogens is weakened, proliferation and differentiation of ISCs is impaired, repair of the intestinal epithelial barrier is weakened, and a large number of inflammatory factors are released. The “triple injury” pattern of intestinal mucosal inflammation is consistent with the clinical characteristics of acute onset, severe intestinal mucosal necrosis, and severe systemic inflammatory response in children with NEC. The occurrence of PC necroptosis is spatially and temporally consistent with NEC events. Therefore, we speculate that necroptosis of PCs is an important event in the pathogenesis of NEC. The dysfunction of PC function and number can lead to the dysfunction of the programmed necrosis pathway “RIPK1-RIPK3-MLKL”, which ultimately contributes to the occurrence of NEC.

### 6.3 Autophagy

Autophagy refers to the isolation of cytoplasmic substances into autophagosomes, which are then fused with lysozyme, resulting in content degradation that can help remove misfolded or aggregated proteins or resolve other intracellular damage (Günther et al., 2013). Similar to apoptosis, autophagy tends to prevent inflammation because the degradation of dead cells occurs in another cell. Autophagy is essential for the development, maintenance, and functioning of PCs (van Es and Clevers, 2014). Since PCs tend to live longer than most other cells in the gut and contain excessive amounts of accumulated proteins that can be recycled by other neighboring cells, autophagy is activated when PCs are damaged or stressed (Günther et al., 2013).

The autophagic gene *Atg16L1* is expressed in IECs (Cadwell et al., 2008). Mice with *Atg16L1* deletion in the intestinal epithelium showed significant defects in the PC granular exocytosis pathway, resulting in reduced antimicrobial peptide secretion. In mice infected with norovirus, *Atg16L1* has a germline gene-trapping mutation, which can further induce morphological and functional defects in PCs (Matsuzawa-Ishimoto et al., 2017; Larabi et al., 2020). Both clinical samples and animal models have suggested that autophagy gene ATG16L1 can promote the severity of NEC by promoting intestinal microbiota-related inflammation and affecting intestinal immune function (Sampath et al., 2017).

Secretory autophagy can also occur in PCs when endoplasmic reticulum (ER) stress leads to phosphorylation of protein kinase RNA-like ER kinase (PERK) (Adolph et al., 2013). p-PERK affects the phosphorylation of initiation factor 2α (ELF2Α), and DC MyD88 is specifically activated by dendritic cells at the TLR signal transduction junction to inactivate Paneth cells, resulting in PC-lysozyme secretory autophagy (Deuring et al., 2014; Yu S. et al., 2020). Stem cells can restore intestinal epithelial tight junction and permeability by regulating ER stress in NEC. (Li et al., 2021).

In conclusion, the specific autophagy that occurs during PC homeostasis is dependent on the microbiome and the proinflammatory cytokine IFN-γ. *Atg16L1* and upstream autophagic *Atg5* have a compensatory role in ER stress as they control the autophagy pathway and help to maintain IEC homeostasis. The disruption of mitochondrial homeostasis in PCs is caused by the synergism of *Atg16L1* deletion and ER stress (Cray et al., 2021).

### 6.4 Pyroptosis

Under stress conditions (such as intestinal ischemia-reperfusion injury) or lipopolysaccharide stimulation, another inflammatory mode of cell death called pyroptosis can occur (Jia et al., 2020). Stress can induce TLR4/NF-κB activation of the NIMA-related kinase 7 (NEK7) promoter region and increase NEK7 expression (Chen et al., 2019). NEK7 interacts with NOD-like receptor pyrin domain-containing protein 3 (NLRP3) and regulates the activation of the NLRP3 inflammasome. These inflammatory bodies act on caspase-8 and convert pro-caspase-1 to cleaved caspase-1 (Fritsch et al., 2019; Patankar and Becker, 2020). Gasdermin D (GSDMD) is cleaved by active caspase-1/4/5/11 and caspase-3 through an intermediate linker, resulting in cytoplasmic swelling and “pore-making activity” (Gaidt et al., 2016; Kovacs and Miao, 2017; Tan et al., 2020). The release of N-terminal fragments of GSDMD, along with inflammatory factors, such as IL-β and IL-18, induces pyroptosis (Huang et al., 2019). Recent studies have shown that the NLRP3 inflammasome is involved in a variety of adaptive immune diseases and that NLRP3 mutations could lead to chronic autoinflammatory syndrome (Yu et al., 2019). The expression of NLRP3 in NEC is significantly upregulated and could stimulate the production of pro-inflammatory cytokines such as IL-1β, IL-6, and IL-18, leading to pyroptosis (Grishin et al., 2016). Intracellular IFN-γ in murine PCs, an important mediator of intestinal inflammation, alters mitochondrial integrity and membrane potential of PCs, leading to a TORC1-dependent pyroptosis distinct from the classical cell death pathway (Araujo et al., 2021). However, the relationship between pyroptosis and NEC remains unclear, which may be a new direction for future research.

## Summary

PCs are a type of IECs that play an important role in maintaining intestinal microbiota homeostasis, ISC growth, and IEC death. Various growth factors secreted by PCs are important for maintaining the intestinal barrier as a defense against pathogens. The loss of PCs causes loss of α-defensin, immunoglobulins, and other products, followed by disruption of the intestinal barrier, thereby leading to NEC. Disruptions of PCs can also reduce the production of lysozyme and other factors that maintain intestinal microbiota homeostasis, resulting in intestinal microbiota disorders and NEC. PCs secrete Wnt3, Notch, and other signaling factors to maintain the function of ISCs and prevent the occurrence of NEC. Autophagy, necroptosis, and apoptosis are also closely related to PCs. Reduced production of antimicrobial peptides by PCs makes the intestine hypersensitive and highly prone to NEC. The development of PCs, intestinal flora homeostasis, and pathogenesis of NEC are closely related; however, only few studies have investigated the possible mechanism of this relationship. Therefore, further exploration of the mechanism affecting the number and function of PCs is of great significance for the prevention, early diagnosis, and treatment of NEC.

## Abbreviations

NEC, necrotizing enterocolitis; PC, Paneth cell; Ig, immunoglobulin; TNF-α, tumor necrosis factor-α; IL-17, Interleukin-17; HD5, human defensin 5; ISCs, intestinal stem cells; NOD2, nucleotide-binding oligomerization domain protein 2; NF-κB, nuclear factor-κB; XIAP, X-linked inhibitor of apoptosis protein; RIPK2, receptor kinase 2; MLKL, mixed-lineage kinase domine-like protein; FADD, Fas-associated protein death domain; PERK, protein kinase RNA-like ER kinase; NEK7, NIMA-related kinase 7; NLRP3, NOD-like receptor pyrin domain-containing protein 3; GSDMD, gasdermin D.
